# The Electrophoretic Revolution in the 1960s: Historical Epistemology Meets the Global History of Science and Technology**

**DOI:** 10.1002/bewi.202200024

**Published:** 2022-09-09

**Authors:** Edna Suárez‐Díaz

**Affiliations:** ^1^ Facultad de Ciencias Universidad Nacional Autónoma de México (UNAM)

**Keywords:** Electrophoresis, experiment, blood diseases, global history, technical objects

## Abstract

This paper uses *zone electrophoresis*, one of the most frequently used tools in molecular biology, to explore two ideas derived from Hans‐Jörg Rheinberger's reflections on experiments. First, the constraining role played by technical objects—instrumentation and material conditions—in the production of knowledge or epistemic things. Second, the production of interconnected experimental systems by such technical objects, which results in the unexpected entanglement of research fields and experimental cultures. By the beginning of the 1960s, the inception of zone electrophoresis in laboratories around the world transformed—some say, revolutionized—the study of proteins. Even today, electrophoresis continues to open research venues and questions in biomedicine, molecular biology, human genetics, and in the field of molecular evolution. In my essay, I seek to look at the interconnected lives of zone electrophoresis and address the broader social, and even global context, in which this apparently humble technique became a salient tool in the production of biological knowledge. In so doing, I aim to take the past and present of the history and historiography of experimental systems to the future, where experiments and technologies are interrogated as they are used in different geographies and contexts, including contexts of poverty.

## 1 Introduction

In 1960, after returning from his graduate studies in Chicago, medical geneticist Rubén Lisker (1931–2015) received a grant of 1,000 Mexican pesos (amounting to less than 100 US dollars) to start a precarious research project on blood types and abnormal hemoglobin in indigenous populations. He also received a small amount from the Mexican *Secretaría de Salud* (Health Ministry) and the local Carnot Laboratories,^1^ but the meager funds did not stop him from pursuing the kind of edgy research that he had become acquainted with at the Michael Reese Hospital in Chicago, Illinois. With the help of a friend, engineer Mauricio Russek‐Bernal from the *Instituto Politécnico Nacional* (*IPN*), he used part of the money to build a small apparatus of paper electrophoresis, one of the new options to perform the decades old technique of separating macromolecules (proteins) by their electric properties and size.^2^ They bought a pair of glass plates, which they then adapted to create an electric power supply with a couple of platinum electrodes and forceps to hold everything together. With this improvised instrument, Lisker managed to perform the first electrophoretic studies of genetic variation in Mexican populations. His primary goal was to identify the same abnormal hemoglobin he had worked with in Chicago, sickle‐cell hemoglobin or HbS for short, as well as the presence of glucose‐6‐phosphate dehydrogenase (G6PD) deficiency, a scarcely understood but prevalent mutation in African and Mediterranean populations. A few studies had been performed before on abnormal hemoglobin in México by Adolfo Karl at the *IPN*, which had not been successful. Nevertheless, in collaboration with long‐time partner Alvar Loria, also at the Hematology Department of the *Instituto Nacional de la Nutrición*, Lisker soon published the first of several papers—nineteen to be exact—on the genetic variation of local populations, reporting several genetic variants in blood proteins. Though he continued to use serological tests and blood‐typing surveys, his first report of a G6PD anomaly used electrophoresis to describe the clinical case of a Sephardic boy, whose family had immigrated from Turkey to Mexico. Indeed, the most common G6PD anomaly, favism, was a disease long associated with Mediterranean and Middle Eastern populations who have fava beans as a common ingredient in local diets.^3^ Later on, using the technical variant of starch gel electrophoresis, Lisker also reported the presence of abnormal hemoglobins—including a *Chiapas* variant—in Mexico's indigenous communities. Lisker's research on blood diseases, which continued until the late 1960s and resumed using new technologies in the 1990s, remains to date one of the most comprehensive studies of the genetics of Mexican populations.^4^

Zone electrophoresis, one of the most frequently used tools in molecular biology laboratories, provides a lens to explore two ideas derived from Hans‐Jörg Rheinberger's extended account of experiments: first, the constraining role played by technical objects—instrumentation and material conditions—in the production of knowledge; and second, the production of interconnected experimental systems by such technical objects, which results in the unexpected entanglement of research fields and experimental cultures. To do so, this essay presents a historical epistemological analysis combined with an original way to see the role of electrophoresis in connecting experimental systems and cultures. I am talking about studies on the globalization of technologies, and the epistemic spaces opened by this type of inquiry. Writing the history of the *uses* of a technology in its different contexts, including contexts of poverty, is a much‐needed task for historians of technology and global history, as David Edgerton has forcefully argued.^5^ To shift the focus to *use* requires us to set aside the idea that technological invention and innovation only take place in a privileged setting and pay attention to the adaptations, applications, and resourcefulness required by the actual historical uses of a technology as it travels beyond its place of origin

As the history of studies on blood proteins shows, knowledge on the mechanisms of inheritance of these variations, the establishment of new molecular variants, and empirical data to feed the theoretical models happened far from the usual centers of knowledge production—Bogota, Mexico City, Baghdad, or Tel‐Aviv. These varied experiments cannot be seen as repetitions of ones performed in metropolitan centers. As part of experimental systems set up in different locations, they were able to generate unexpected results and authentic surprises.^6^ Moreover, I seek to look beyond the *innovation* of zone electrophoresis in protein research laboratories in the United States and Western Europe, opting to address the broader social, and even global context, in which this once sophisticated but now apparently humble technique became a salient tool in the production of knowledge. Zone electrophoresis thus can be seen as a material node that tied together the experimental cultures of protein chemistry, human genetics, biomedicine, and evolutionary biology, but also incorporated networks of scientists worldwide, as molecular biology struggled to show its relevance for understanding disease and global medical interventions not just in industrialized countries but as part of the developmentalist agenda. In paying attention to these locations, I aim to engage with the present and future of the history and historiography of the life sciences, where historical epistemology and the global history of science and technology can fruitfully complement each other.

## 2 Electrophoresis after the Second World War

The clear theoretical implications for physical chemistry and extensive biomedical and military applications during and after WWII are exemplified in Angela Creager's essays on Edwin Cohn's research.^7^ The solution‐based or moving‐boundary method of the Tiselius apparatus developed in the 1930s and 1940s, however, was not only difficult to operate and voluminous, but also expensive and beyond the capacities of most laboratories around the world.^8^ By contrast, the electrophoresis apparatus that today inhabits any molecular biology or biochemistry lab is a product of the postwar industrialization of biomedical research. In the decades following the war, the industrial manufacture of research instruments expanded massively in the continued efforts to produce functional, compact, and affordable instruments that would meet the specific needs of the growing community of researchers across the globe.^9^

Historian of science Lily Kay recounts that by the end of the war in 1945, Klett Industries debuted a compact model of the costly Tiselius apparatus, the antecedent of modern zone electrophoresis. Costing 4,000 USD (compared to the 6,000 USD price tag of the original apparatus).^10^ Perhaps more important than its reduced price, was the fact that this new device did not require the intensive work (and salary) of a specialized technician to operate it (about 5,000 USD per year).^11^ By 1950, at least four different bench models existed that were manufactured and commercialized by Perkin‐Elmer at the lower, but still far from universally affordable, cost of 3,000 USD, approximately 35,000 USD in today's dollars.^12^ These new instruments saved precious laboratory space and required only a one‐week training course before they could be put to use. With the arrival of radioisotopes and scintillation counters in the 1950s, electrophoresis became a relatively accessible though still sophisticated instrument available in laboratories in the United States and Europe.

The pervasiveness of electrophoresis in experimental biology hit its highest peak, however, during the next decade. At the beginning of the 1960s, the development of zone electrophoresis, also called *molecular sieving electrophoresis* because of its ability to segregate protein molecules in a sustaining material, radically altered the availability of the method. Contemporary scientists and historians alike, have labeled the introduction of the new technology a “revolution” in the study of proteins.^13^ Historian of science and medicine Howard Chiang has reconstructed the consistent search for a sustaining medium that would allow the easy and complete molecular separation and visualization of proteins using the principles of electrophoresis. Silicon and agar gels, filter paper, pectin gel, cellulose acetate, and starch gel were tested, used, and eventually discarded, until the choice of polyacrylamide gel standardized the material universally present in today's laboratories. However, as Chiang argues, this was not a simple test and error enterprise: it involved efforts and knowledge from scientists in different fields of research, including physical chemistry, immunology, and biochemistry, all of which had something to say about the properties and behavior of proteins.^14^

The establishment of starch gel and polyacrylamide gel electrophoresis, at the end of the 1950s and the beginning of the 1960s, made possible the explosion of applications beyond medicine and biochemistry, in the closely related field of population genetics. Here, the study of human evolution and migration, and of the effects of atomic testing on genetic mutations, converged on the attention to “primitive populations” in contemporary parlance.^15^ Population genetics was, indeed, a hot field during those years, linking the study of disease and evolution as two faces of the same quest: the study of genetic variations, and their measurement made possible by zone electrophoresis. In 1966, Harry Harris, Galton Professor of Human Genetics at King's College, published one of three papers considered crucial to contemporary theoretical debates in population genetics, at a time when Cold War anxieties surrounding atomic weapons testing could not be higher.^16^ In parallel, for countries in the so‐called Third World and for segregated African‐American populations in the Southern United States, electrophoresis enabled the assessment of genetic abnormalities in blood proteins, and became instrumental for those promoting the molecular approach to disease, encapsulated in the concept of “molecular disease.”^17^ The study of “blood diseases in the backyard,” as I have previously referred to this kind of research, linked several of those contemporary preoccupations.

Ruben Lisker, like many other geneticists and medical anthropologists around the world, participated in the international and regional networks organized by the World Health Organization and the Pan American Health Organization round those interrelated topics. They were part of scientific committees created to produce global knowledge and international health policies in the twin fields of human genetic variation and the molecular understanding of medical conditions; at the same time these studies were hailed as an early example of the uses of pharmacogenetics.^18^ Blood diseases, including sickle cell anemia, different types of thalassemia, and G6PD deficiencies (one of which is related to favism), targeted populations in Africa, the Mediterranean, East Asia and Latin America, thus becoming a de facto biomedical enterprise alternative to the war on cancer. The extensive network of human geneticists surveying molecular abnormalities, most of them using starch gel electrophoresis, has caught the attention of historians of science, though it deserves to be further explored.^19^

## 3 The Interconnected Lives of Electrophoretic Experiments

**Figure 1:**
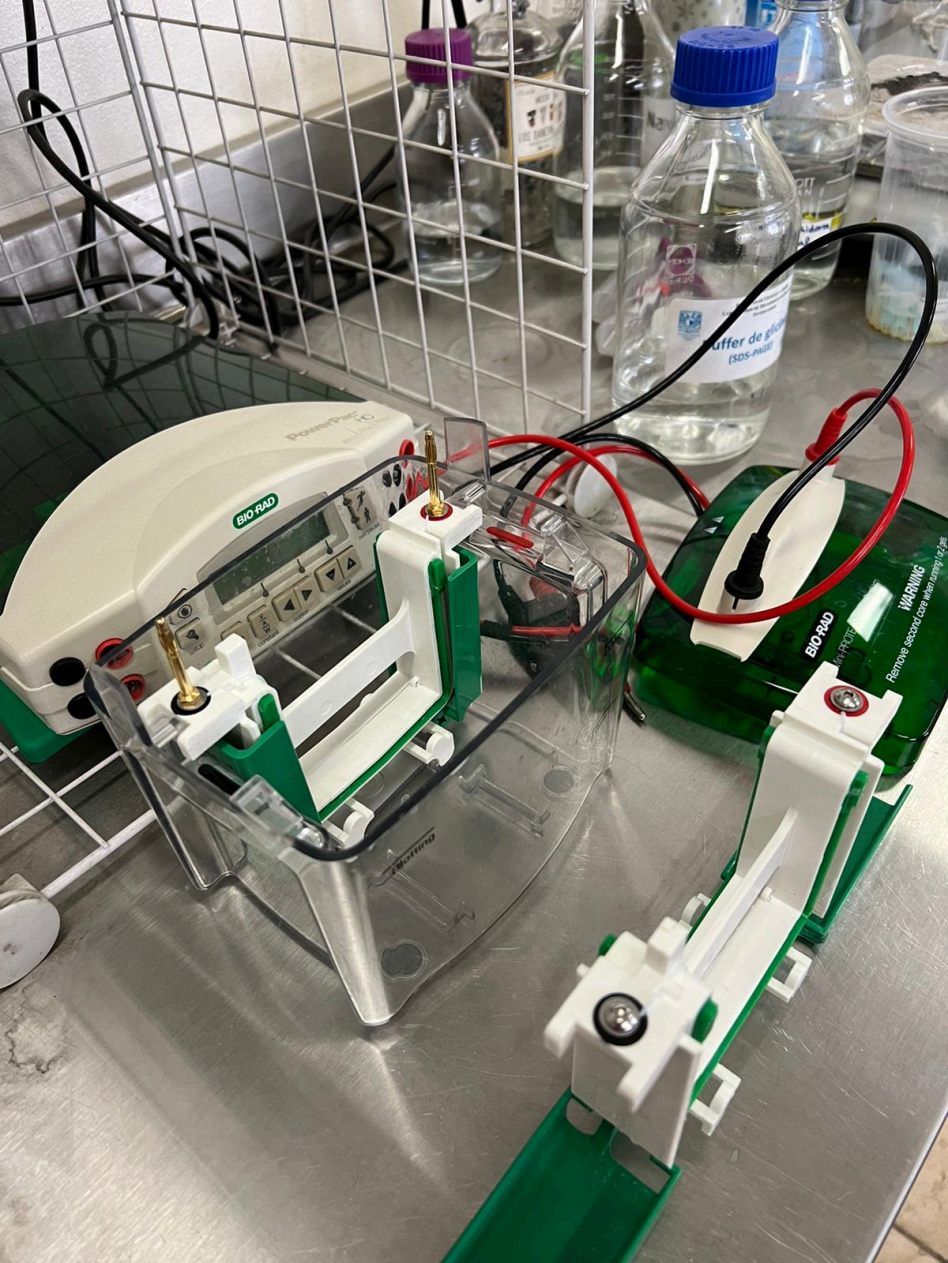
Bio‐Rad electrophoresis apparatus and power supply at the author's brother laboratory of plant molecular biology in Mexico City, 2022. Photograph taken by the author's brother, Javier Andrés Juárez‐Díaz.

In contrast to other big manufacturers of research equipment,^20^ the main manufacturer of electrophoresis equipment and consumables in today's laboratories is a US‐based family firm, Bio‐Rad Laboratories, Inc. The company was founded in 1952 in Berkeley, California, and entered the blooming experimental culture of identification, separation, purification, and analysis of chemical and biological materials. Bio‐Rad first made a name for itself in the field of analytical chemistry through ion exchange resins. In 1964, the firm introduced “gel chromatography media […] for separating proteins by molecular weight,” thus saving preparation time for scientists.^21^ The company has created an empire in the manufacturing of equipment for life science research and medical diagnostics, and in the last decades has additionally absorbed other industries in the agricultural, chemistry, and biomedical fields. Even today, electrophoresis continues to open research venues and solve relevant questions in biomedicine, molecular biology, human genetics, and molecular evolution. Bio‐Rad equipment is found in laboratories around the globe (Figure 1), most probably because the technique provides one of the easiest procedures to *visualize* the properties of proteins and nucleic acids. The interaction of the mass and electric properties of a macromolecule with the buffer and the supporting material in an electric field is manifested in the characteristic bands—“traces,” in Rheinberger's conceptualization. These traces constitute the elementary material manifestation preceding a scientific representation.^22^ Those traces, however, only become data in their comparison with one another; it is only in the comparative alignment of bands that anomalies (variations) can be incorporated in the mathematical or in the mechanistic models of theoretical population genetics, protein chemistry, genetics, and biomedicine.

What is, precisely, the place of electrophoretic apparatuses and methods in those different but connected research fields? The technological intervention exerted by electrophoresis on blood materials results in traces that take on one of two roles depending on the specific experimental culture: *measuring*, as in measuring the molecular weight or the electric valance of a protein, or *identifying biological phenomena*, as in providing the trace for an abnormal or variant protein. An electrophoresis experiment also provides a more complex type of measurement, or calculation, as it delivers the traces used *to infer* the amount of genetic variation within a population. In these three different cases, electrophoresis participates in fields of research as different as protein chemistry, molecular biology and biomedicine, and theoretical population genetics, respectively. The epistemic role it plays is also different: identifying biological phenomena—like an abnormal hemoglobin—is a classic case of the laboratory sciences, while providing measures of genetic variation, according to Richard Lewontin, was required to meet the chronic lack of empirical data “to feed the theoretical machine” of mathematical population genetics.^23^ This latter role is close to the traditional view of the place of experiments in the philosophy of science, namely, providing empirical evidence to test a hypothesis. At the same time, the history of the contentious debate on the amount of genetic variation in natural populations that followed from these experiments, gives credence to sociological accounts on the difficulties of experimental closure. In 1966, electrophoresis was supposed to revolutionize the whole field of human genetics, but in fact the polemic results only amplified the debate and ended up challenging the very roots of evolutionary theory.^24^ This case shows, nevertheless, the fundamental role the seemingly humble technology of electrophoresis played in linking protein chemistry with the preoccupations of biomedicine and human genetics, as it had been promised and promoted by the protagonists in these developments.

## 4 Concluding Remarks

After the war, electrophoretic apparatuses benefited from the industrialization and commercialization of research instruments, but also from its low relative cost, ease of use, compact size, and mobility—though one should not take for granted the availability of constant electric power supply in many areas of the world in the 1960s and even today! Zone electrophoresis became an affordable and relatively mobile technology. New industries like Bio‐Rad, Perkin‐Elmer (once the parent company of Applied‐Biosystems), and many others, were created, fused, dismantled, or remained relevant and present in today's laboratories, literally around the world. The study of the design, marketing, and distribution of these technologies internationally is by itself a topic for global historians of technology, not reducible to questions of translation or communication, much less to the metaphor of circulation.^25^

For historians of science, and in particular the life sciences, the topic of the global interconnection of experiments is a necessary extension of the *practice turn* of the 1990s and of the conceptualization of experimental systems as the material conditions that enable the generation of knowledge. Indeed, experiments have never been the same for historians and philosophers of biology—especially for those interested in molecular biology—after Rheinberger's early 1990s publications on the *historiality* of experimental systems.^26^ “Experiment, Difference, and Writing (I and II),” published back‐to‐back in 1992, and followed by *Toward a History of Epistemic Things* ^27^ were particularly influential and opened a host of debates concerning, among other topics, the indeterminacy of experimental systems, technological determinism, and the place of the broader historical context in Rheinberger's microscopic analyses of experimental practices. Among the many themes introduced in his tireless academic trajectory, the dissection of experimental systems has stood the test of time, and his ideas have been surgically analyzed, transformed, and entangled with novel areas of research in the history and philosophy of science and historical epistemology. Most recently, Rheinberger has extended his fine‐grained dissection of experiments and experimental systems in his book *Spalt und Fuge. Eine Phänomenologie des Experiments*.^28^ Rheinberger's argument aimed, in his words, “to avoid some of the pitfalls of social constructivism with respect to the objects of science,” by placing “the genesis and development of scientific facts […] in the relation between objects themselves.”^29^

The same argument is valid in addressing the globalization of knowledge production, a process that cannot be reduced to the mere recognition of the asymmetrical relations between scientific centers, or to the extension of colonial or postcolonial exchanges. During the 1960s, a period also known as the “decade of development,” electrophoresis became one of the most popular and used technologies to survey so‐called primitive populations. This included the study of groups of people resisting and participating in liberation wars, as was the case when the US Army studied the genetics of the Vietnamese populations for malaria resistance and other biomedical conditions of interest for the effectiveness of the military enterprise.^30^ Moreover, grounding of the production of knowledge in a global context directs us to recognize the *epistemic injustice* involved in these transactions, a concept developed in feminist philosophy to account for the downgraded status of an epistemic subject.^31^ In this case, the contributions of scientists in regions outside Western Europe and the United States have been discriminated and marginalized, and wrongly perceived as epistemically lesser despite their valuable contributions to knowledge of human genetics. The globalization of zone electrophoresis and its many uses across disciplinary borders and geopolitical lines, remains a fabulous task for those interested in the broader meaning and implications of the interconnected lives of experiments.

## Footnotes

n1 Created in 1941, in Mexico City, *Laboratorios Carnot* was one of the few pharmaceutical laboratories in Mexico committed to scientific research and technological innovation in Mexico. In 1965, Lisker was awarded the Carnot Prize by the National Academy of Medicine for his surveys of indigenous populations.

n2 Kay 1988.

n3 On the relevance of favism in the study of human population genetics in the Middle East, see Burton 2021, chapter 5. Lisker, like many of the geneticists studied by Elise K. Burton, was also an Ashkenazi Jew whose family had migrated from Russia to the United States and ended up in Mexico.

n4 In his long career, Lisker worked also on lactose‐deficiency in Mexican populations and was an early researcher of bioethics. See Suárez‐Díaz 2014 and Suárez‐Díaz 2017.

n5 Edgerton 2010 and Edgerton 2007. See also Schäfer and Valeriani 2021.

n6 Rheinberger 1997.

n7 Creager 1998 and Creager 1999.

n8 Kay 1998, on 51, 67, and 69–70.

n9 The production of laboratory technologies and the specific tasks of the scientists also opened the door to new experimental designs. The close interaction between manufacturers and scientists is recounted in detail in Rheinberger's account of the history of the liquid scintillating counter. Rheinberger 2001. Also, see Alexander von Schwerin's article in this volume.

n10 Kay 1988, on 69.

n11 Ibid., on 51.

n12 Ibid., on 69.

n13 Gottlieb 1971 and Vesterberg 1989 offer personal accounts. Dietrich 2006 also calls attention to the revolutionary effect of electrophoresis in population genetics.

n14 Chiang 2009.

n15 See, for instance, World Health Organization 1964. There is ample historical literature on the subject, including: Ventura‐Santos et al. 2014; Radin 2017; Suárez‐Díaz 2019.

n16 Harris 1966. In 1965, Harris was appointed Galton Professor of Human Genetics and head of the genetics and biometry department at University College in London. From 1961–1976, Harris also directed the human biomedical genetics unit of the Medical Research Council. The other two papers are: Hubby and Lewontin 1966; Johnson et al. 1966. On genetics and atomic testing, see: Beatty 1987; Lindee 2008; de Chadarevian 2006; Hamblin 2007. For the impact of these experiments on evolutionary studies, see: Dietrich 1994; Suárez‐Díaz and Barahona 1996.

n17 Wailoo 1999, on 154–159; Wailoo 2015; Suárez‐Díaz 2017. On the study of blood anomalies by Middle‐Eastern geneticists, see Burton 2018 and Burton 2021.

n18 For instance: World Health Organization 1966; Pan American Health Organization 1968.

n19 De Souza and Ventura Santos 2014; Suárez‐Díaz 2014; Suárez‐Díaz 2017; Burton 2018. An important addition to this literature is Burton 2021.

n20 See von Schwerin's article on the early history of Applied Biosystems in this volume.

n21 Bio‐Rad, “Our History,” online: https://www.bio‐rad.com/en‐de/corporate/our‐history?ID=MR8ISY4VY (accessed 17 April 2022).

n22 Rheinberger 2021, chapters 1 and 3.

n23 Lewontin, 1974.

n24 Lewontin 1991; Dietrich 1994; Suárez‐Diaz and Barahona 1996.

n25 Mateos and Suárez‐Díaz 2019.

n26 Rheinberger 1992a; Rheinberger 1992b.

n27 Rheinberger 1997.

n28 Rheinberger 2021.

n29 Rheinberger 2005, on 408. Rather contradictorily, Rheinberger has been accused of both technological determinism and idealism. See Bloor 2005.

n30 Frischer et al. 1973. I thank Elise K. Burton for providing me this reference in relation to our common interest on James E. Bowman's work around the world.

n31 I thank Lara Keuck for her referral to the original literature on this important concept. See Fricker 2007 and Fricker 2017. More recently, the concept of epistemic injustice has been mobilized and debated in the context of international public health and the COVID‐19 pandemics, see Bhakuni and Abimbola 2021.
